# Reducing the global burden of Preterm Birth through knowledge transfer and exchange: a research agenda for engaging effectively with policymakers

**DOI:** 10.1186/s12978-016-0146-8

**Published:** 2016-03-18

**Authors:** Gavin Yamey, Hacsi Horváth, Laura Schmidt, Janet Myers, Claire D. Brindis

**Affiliations:** Duke Global Health Institute, Duke University, Durham, North Carolina USA; Global Health Sciences, University of California, San Francisco, San Francisco, California USA; School of Medicine, University of California, San Francisco, San Francisco, California USA; Division of Prevention Science, School of Medicine, University of California, San Francisco, San Francisco, California USA; Philip R. Lee Institute for Health Policy Studies, University of California, San Francisco, San Francisco, California USA

## Abstract

Preterm birth (PTB) is the world’s leading cause of death in children under 5 years. In 2013, over one million out of six million child deaths were due to complications of PTB. The rate of decline in child death overall has far outpaced the rate of decline attributable to PTB. Three key reasons for this slow progress in reducing PTB mortality are: (a) the underlying etiology and biological mechanisms remain unknown, presenting a challenge to discovering ways to prevent and treat the condition; (ii) while there are several evidence-based interventions that can reduce the risk of PTB and associated infant mortality, the coverage rates of these interventions in low- and middle-income countries remain very low; and (c) the gap between knowledge and action on PTB—the “know-do gap”—has been a major obstacle to progress in scaling up the use of existing evidence-based child health interventions, including those to prevent and treat PTB.

In this review, we focus on the know-do gap in PTB as it applies to policymakers. The evidence-based approaches to narrowing this gap have become known as knowledge transfer and exchange (KTE). In our paper, we propose a research agenda for promoting KTE with policymakers, with an ambitious but realistic goal of reducing the global burden of PTB. We hope that our proposed research agenda stimulates further debate and discussion on research priorities to soon bend the curve of PTB mortality.

**Electronic supplementary material:**

The online version of this article (doi:10.1186/s12978-016-0146-8) contains supplementary material, which is available to authorized users.

## Background

Preterm birth (PTB)—birth before 37 weeks of gestation—is the world’s leading cause of death in children under 5 years. In 2013, over one million out of six million child deaths were due to complications of PTB [1]. The rate of decline in child death overall has far outpaced the rate of decline attributable to PTB. While the average rate of all-cause, under-five mortality fell by over 4 % each year between 2000 and 2013, the death rate from prematurity fell by only an average of about 2 % each year over the same time period [1].

There are three key reasons for this slow progress in reducing PTB mortality. First, while many risk factors for PTB have been identified (Table 1), the underlying etiology and biological mechanisms remain unknown [2]. This lack of knowledge presents a major challenge to discovering ways to prevent the condition and to treat those infants affected by PTB. Second, while there are several evidence-based clinical and population-based interventions that can reduce the risk of PTB and associated infant mortality, the coverage rates of these interventions in low- and middle-income countries (LMICs) remain very low [2]. Finally, the gap between knowledge and action on PTB—the “know-do gap” [3]—has been a major obstacle to progress in scaling up the use of existing evidence-based child health interventions [4], including those to prevent and treat PTB [5].

**Table 1:** PTB risk factors, prevention, and management. Evidence is summarized in reference [2]

The physiological mechanisms behind PTB remain largely unknown, although a number of risk factors have been described. Medical risk factors include infections, non-communicable diseases and their risk factors (diabetes, hypertension), and multiple pregnancies; social risk factors include low or high maternal age, poverty, and receiving antenatal care for the first time at a late stage in the pregnancy; and behavioral risk factors include tobacco, alcohol, substance use, and stress.
The *Born Too Soon* report summarized the key evidence-based interventions for preventing PTB and for reducing mortality among those born prematurely [2].
Prevention focuses on prenatal care (e.g., education, nutrition, treatment of sexually transmitted infections, family planning); antenatal care; obstetric care; and policies to tackle risk factors, such as smoking in pregnancy.
Reducing mortality focuses on newborn care (e.g., feeding, thermal care); kangaroo care; neonatal resuscitation if needed; management of complications; and neonatal intensive care, if needed.
Managing preterm labor can both prevent PTB and reduce mortality among premature babies. Such management includes antenatal corticosteroids; antibiotics for premature rupture of the membranes; and tocolytics to slow down preterm labor.
Broader social, financial, agricultural and other policies that are being investigated for their potential role in reducing the burden of PTB include measures to improve household food security; conditional cash transfers to increase patient uptake of services; and performance-based financing to improve quality of care.

The evidence-based approaches to narrowing this gap have become known as knowledge transfer and exchange (KTE). Such exchange involves the “synthesis, exchange, and application of knowledge by relevant stakeholders” in ways that accelerate the benefits of global and local innovation in health [3]. KTE works best when it is nuanced in its application and customized to the needs of its stakeholders.

In PTB, the stakeholders are a diverse group that includes mothers, fathers, and their babies; doctors, nurses and other health care providers; community-based organizations; people researching the causes of PTB and new ways to prevent and treat its effects; and policymakers. These policymakers are at local, national and international levels, including, for example, individuals creating and enacting health policy in health departments, ministries of health, and international health agencies. Policymakers are critical to enacting the ambitious but realistic goal of reducing the global burden of PTB because they influence the way resources are used to leverage the evidence or the “know” – they are key to closing the know-do gap because they have control over the resources that make enacting the evidence possible. In this paper, we review the literature and recommend a KTE research agenda that could help policymakers close these gaps to reduce the burden of PTB.

We begin by examining the state of the current evidence on what works best in KTE with policymakers, and on the barriers and facilitators to successful uptake and use of evidence. We then propose a framework for categorizing where the KTE know-do gaps are positioned in the evidence-to-policy cycle. Finally, we end by making recommendations for researchers seeking to close the gaps in order to reduce the burden of PTB.

## KTE with policymakers: a global imperative for reducing PTB

Over the past few decades, KTE has emerged as a framework to optimize the translation of the best research evidence and authentic stakeholder perspectives into better health outcomes [6]. KTE with policymakers in particular is a critical yet understudied area of research. The intended outcome of KTE is evidence-informed policy-making (EIP), which can improve health outcomes by bringing the best-available evidence into health policy decision-making processes [7]. Greater adoption of evidence into policies is associated with a shift away from biased, wasteful and irrelevant health policy decisions, which are too often taken with poor consideration of local conditions [3]. KTE moves us toward a more transparent, systematic approach, informed by locally-applicable evidence and made with appropriate engagement of all stakeholders [8].

This kind of shift is urgently needed to reduce the burden of PTB. Until recently, PTB was a very low priority on the global health agenda. However, the new Sustainable Development Goal (SDG) for Health, SDG 3, calls for “an end” to avertable child and neonatal deaths by 2030 [9]. SDG 3 is one of the 17 SDGs adopted by UN member states in September 2015. Without addressing PTB, such bold post-2015 goals will be impossible to reach [10]. As Table 1 illustrates, a range of evidence-based policies could substantially reduce PTB mortality if they were truly marshalled. These policies may target risk factors, such as policies to reduce smoking in pregnancy, as well as the accessibility, costs and quality of maternal and newborn care, such as policies to scale up kangaroo care.

Evidence of successful KTE with policymakers would include increased rates of adopting evidence-based interventions (e.g., higher rates of kangaroo baby care, adoption of stricter tobacco control policies, and increased implementation of antenatal community outreach services in LMICs). Successful KTE should also mean that local knowledge—that is, evidence that is shared by members of the affected community— is fully integrated in the decision-making and reflected in policy outcomes.

## Empirical research on improving KTE with policymakers

### Facilitators and barriers to KTE

A 2014 systematic review of studies examining facilitators and barriers to the use of evidence in health policymaking found 145 separate studies, including 13 systematic reviews [11]. The most commonly identified factors that facilitate or obstruct the use of scientific evidence by policymakers are summarized in Table 2. Most facilitating factors relate to optimizing the usability of research findings: making research evidence easily accessible to policymakers, for example, through close collaborative relationships with researchers. Commonly identified barriers to the uptake of evidence by policymakers reflect the opposite: the greatest barriers are limited access to research evidence and the lack of “research literacy” among policymakers.

**Table 2:** Facilitators and barriers to KTE with policymakers

A 2014 systematic review of studies examining facilitators and barriers to the use of evidence in health policymaking identified 145 studies, including 13 systematic reviews [11]. The most commonly identified facilitators to the use of scientific evidence by policymakers are listed below (*n* refers to the number of studies that reported any given factor):
• Good availability of and access to research and improved dissemination of research (*n* = 63 studies)
• Strong collaboration between researchers and policymakers (*n* = 49)
• Clear, relevant, and reliable research findings (*n* = 46)
• Strong personal relationships between researchers and policymakers (*n* = 39).
The most commonly identified barriers to the uptake of evidence by policymakers were:
• Poor availability of and access to research and poor dissemination of research (*n* = 63)
• Unclear research findings of little relevance and poor reliability (*n* = 54)
• Evidence not available at the time when policymakers needed it most, i.e., the windows of opportunity for getting evidence into policy were missed (*n* = 42)
• Lack of research skills among policymakers (*n* = 26)
• Economic costs involved in dissemination activities (*n* = 25).

### Two key approaches to KTE with policymakers

To our analysis of barriers and facilitators to KTE implementation, we added an analysis of existing approaches to EIP. We discovered multiple interventions that hold promise for efficiently and effectively transferring and exchanging knowledge about solutions to PTB with policymakers. These clustered into two groups, which we label “linear” and “political economy” approaches for tackling the problem.

**Fig. 1:**
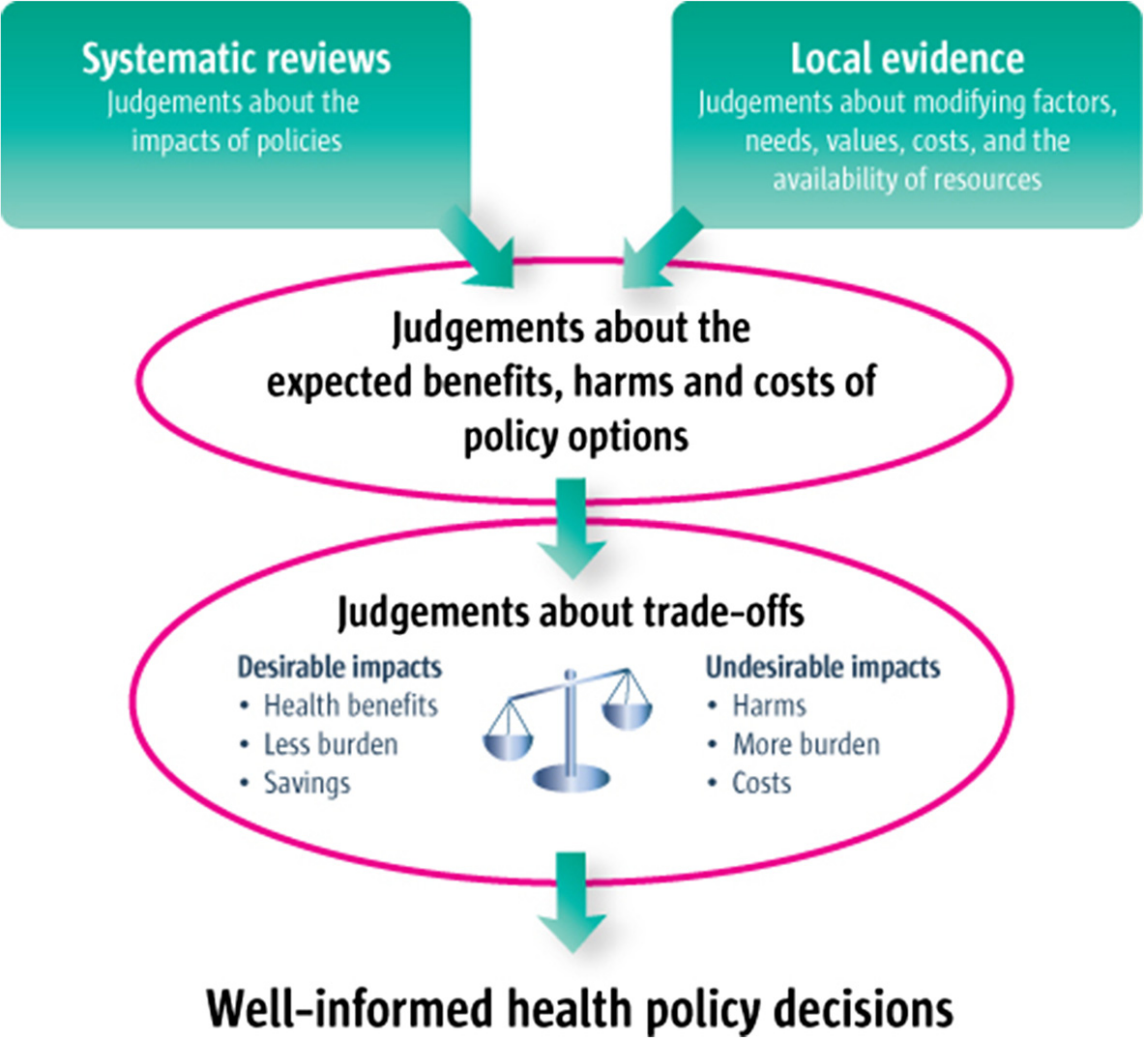
A “linear” evidence-informed policymaking approach (figure from [8])

The “linear” EIP approach, also known as “getting research into policy” or GRIP, starts from the viewpoint that there is a gap between the generation of health evidence on PTB and practical policymaking; closing this “evidence to policy” gap is an important tool for health improvement [8]. The linear approach assumes that there is a series of steps—a linear pathway—from generating research evidence to evidence-informed policymaking (Fig. 1). This approach is supported by empirical evidence showing that clear, relevant, and reliable research findings facilitate the use of evidence by policymakers [11]. An example of a specific KTE strategy flowing from this approach is the production by researchers of a short, accessible evidence brief targeting policymakers that summarizes the results of a systematic review and discusses potential policy implications [12].

With a linear approach, certain forms of evidence, particularly systematic reviews and randomized controlled trials (RCTs), are given high priority, since they are the most reliable forms of evidence and are least prone to bias. They are thus positioned at the top of the “hierarchy of evidence” [13]. Here, the relevant research evidence on PTB would be collated and appraised, implications for potential policy directions developed, the evidence would be used to make judgments about the pros and cons of particular policy options, and then it would “transferred” to policymakers who are primed to follow up.

This approach would recognize that PTB policymaking demands different kinds of input from researchers at different stages in the policy process. It also raises the possibility of earlier engagement of policymakers by researchers so that researchers have a better sense of the types of issues of most concern to their audience. Early on, policymakers often want a menu of options, typically with specific information on the pros and cons of each option. Later in the process, they may want technical assistance in drafting and implementing specific policy choices.

A second approach, the “political economy” approach, sees both the research process itself and the transfer of research evidence to policy as heavily influenced by competing economic interests, social values, and power dynamics [14, 15]. These forces shape the particular research questions that receive attention and funding, not to mention if and how scientific evidence from funded research is brought to bear on the decision-making process. In this approach, policymaking cannot be reduced to a series of linear, technically oriented steps because external social pressures are such powerful drivers. As Barnes and Parkhurst argue in their critique of linear evidence-to-policy processes, “decision-making about global health policy fundamentally involves making choices between, and allocating resources towards, competing alternatives, which have different values to society” [14].

The political economy perspective ultimately calls for a participatory approach to decision making. The very process of generating scientific evidence, and bringing it to bear on policy decisions, should involve “setting up a democratic and deliberative approach, which is inclusive, explicit, transparent, and accountable to the needs of the people these decisions affect” [14]. Key components include [14, 15]:

- using multiple forms of evidence, not just systematic reviews (e.g., including the “lived experience” of the populations affected by the policy);
- acknowledging and understanding the political processes, vested interests, and ethical dimensions that shape policymaking;
- encouraging scientists and experts to support “open reflection and debate” about the evidence; and
- encouraging policymakers to routinely question their own roles, relationships, and values in relation to their use of research evidence—for example, by reflecting on whether they include, exclude, or perhaps do not “hear” different bodies of evidence.

### Evidence on the effectiveness of different KTE strategies targeting policymakers

Our review of the evidence (summarized in Additional file 1) suggests that empirical research documenting the effectiveness of KTE with policymakers is sparse. To date, there have been no relevant randomized trials of KTE interventions for policymaking. There have, however, been multiple non-randomized studies of KTE strategies based mostly on the linear EIP approach, and multiple systematic reviews of these studies. Six strategies based on a linear approach that have shown effectiveness are summarized in Table 3 [6–8, 16, 17] (Additional file 1).

**Table 3:** Effective KTE strategies with policymakers, based on the linear EIP approach

There is evidence from systematic reviews to show that the following six strategies can increase health policymakers’ “intention to act” on the evidence presented to them [6–8, 16, 17] (Additional file 1):
• Providing policymakers with evidence briefs: short, accessible summaries of systematic review and local evidence, describing the context, problem and policy options, and paying attention to issues such as policy implementation, equity, local applicability and the quality of the underlying evidence.
• Deliberative dialogues: these are in-person discussions between researchers and policymakers, typically followed by a year-long service in which policymakers receive evidence updates; the dialogues are based on evidence briefs.
• Providing policymakers with systematic review-derived products: summaries of reviews, overviews of reviews, and policy briefs.
• “One-stop shops” of optimally-packaged online systematic review-derived products. An example of a one-stop shop is *Health Evidence* (www.healthevidence.org), which allows users to find evidence on the effectiveness of public health interventions; the resource can be searched by topic (e.g. premature birth, maternal depression, etc.).
• “Rapid response units,” which provide policymakers written summaries, telephone consultations or in-person consultations about best evidence.
• SUPPORT tools for evidence-informed health policy making. A set of tools developed by the Supporting Policy Relevant Reviews and Trials (SUPPORT) project aimed at helping decision makers in health to make decisions informed by evidence. The tools cover topics such as identifying evidence needs, finding the evidence, and applying the evidence.

Our review also identified two key KTE strategies with policymakers based on a political economy approach: (i) the collective impact strategy, which has been used to address conditions such as nutritional deficiencies, communicable diseases, obesity, and substance misuse [18–21], and (ii) learning collaboratives, which are aimed at creating sustainable learning partnerships amongst policymakers and researchers [22–24]. The existing evidence on the effectiveness of these two strategies is summarized in Table 4.

**Table 4:** KTE strategies with policymakers based on a political economy approach

Strategy	Outcome	Type of supportive evidence
Collective impact: a collaborative, multi-sectoral approach to achieving policy change, with five characteristics: “a common agenda; shared measurement systems, mutually reinforcing activities, continuous communication, and the presence of a backbone organization” [21]	Reductions in a wide range of health-related outcomes, e.g. obesity, substance use, nutritional deficiencies [18–21]	While“evidence of the effectiveness of this approach is still limited” [21], there are now multiple large case studies suggesting that multi-sectoral collective impact approaches can have a larger effect than working in isolation [18–21]
Learning collaboratives: these bring policymakers together in an ongoing way to share knowledge about how to improve a specific health outcome. Common characteristics of learning collaboratives are: • An explicit mission • Routine learning activities (e.g. continuous learning groups) • Relationship-building (e.g. through social networking)	Modest benefits in improving quality of care	A systematic review identified 9 studies using a controlled design (two were RCTs); these measured the effects of collaboratives on care processes or care outcomes. The evidence for quality improvement was “positive but limited and the effects cannot be predicted with great certainty” [24]. Other case studies have suggested positive outcomes [22, 23].

## Proposed research agenda for engaging policymakers in KTE

### Underlying principles

**Fig. 2:**
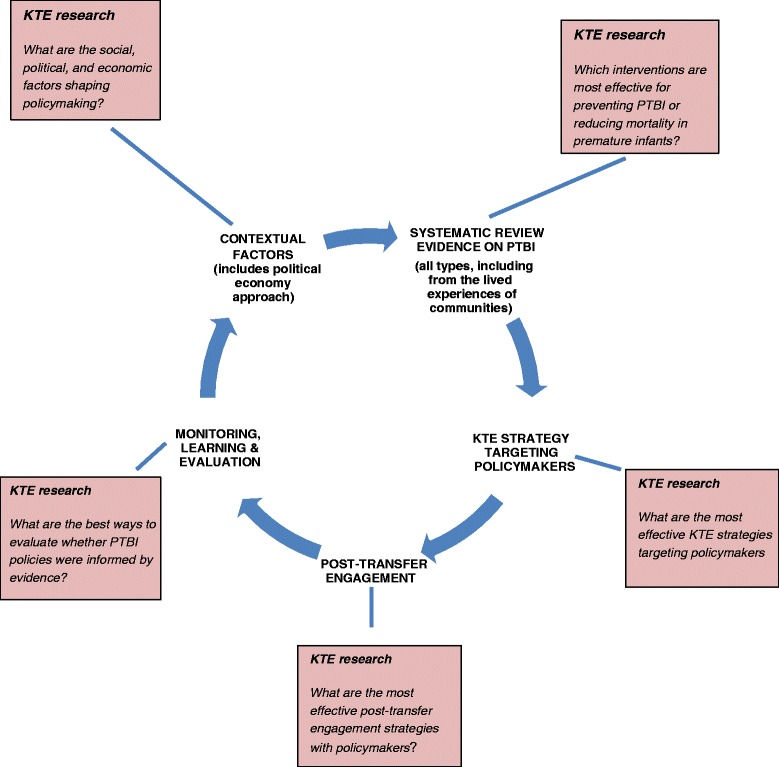
KTE research framework for reducing the burden of PTB—targeted at engaging policymakers

Based on our literature review and analyses described above, we developed a proposed framework to guide consideration of unanswered research questions about the KTE process for policymaking on PTB. This framework, summarized in Fig. 2, is premised on two general principles:

- *Context matters*. KTE never happens in a social, political, and economic vacuum. For example, the capacity for policymaking will vary according to all aspects of the local environment, including the economic, financial, regulatory, and social environments, which can vary at both local and country levels. For these reasons, effective KTE cannot follow a “one size fits all” approach. A key avenue of research will be to study how the political, economic, cultural and social environments in targeted areas come into play in designing effective KTE interventions and policies to enact them.
- *Effective KTE does not stop once the knowledge has been transferred*. Transferring research evidence is a necessary but insufficient condition for achieving sustainable impacts on PTB. Sustained follow-up, continued engagement with policymakers, and tracking the fidelity of the evidence-to-policy process is likely needed to achieve optimal outcomes. Thus, a key avenue of research should be to study the required duration of researcher engagement needed to support “stickiness” and sustainability of knowledge in policy formation, implementation, and short and longer term outcomes.

### Proposed framework for KTE targeting policymakers

Our proposed KTE research framework incorporates both the “linear” and “political economy” approaches to evidence-informed policymaking. Its five components include: (1) understanding the context for policymaking; (2) systematically reviewing the evidence that needs to be transferred in policymakers; (3) using the best-available KTE strategies for transferring that evidence; (4) ongoing, post-transfer engagement; and (5) evaluation of whether evidence shaped policy.

The circular design of the figure underscores the importance of feedback loops and integration of the two approaches. We recognize that the transfer and exchange of evidence to and between policymakers is unlikely to follow the neat, step-by-step pattern and the sequence of steps implied by Fig. 2 (e.g., sometimes research is conducted after a policy has been instituted). And while the target audience is clearly policymakers, Fig. 2 acknowledges that other actors and stakeholders, including families, community groups and health providers, must be part of the process.

## Research questions and methods to optimize KTE with policymakers

Using our KTE research framework, we offer an initial “long list” of research questions that could guide refinements in KTE strategies focused on PTB. We also prioritize those likely to be the most important and impactful, and then we propose appropriate methodological approaches to tackle each these questions (Table 5).

**Table 5:** Research questions related to improving KTE with policymakers

Goal of the research	Research question	Examples of studies in different contexts:
Understanding and improving elements of the context in which KTE occurs	What are the political, economic, cultural, and social contextual factors that influence PTBI policymaking?	Compare and contrast the influence that key stakeholders have had in PTB policy formation and implementation in developing countries (for example, Kenya, Uganda) to those in underserved communities in developed countries (for example, Fresno, California). Understand how the social context has influenced the degree to which stakeholder groups are able to influence policy formation.
	What are the specific barriers and facilitators to the uptake of evidence by policymakers in the research site under study?	Qualitative research with policymakers in the research site under study; document analysis; case studies
	In the site being studied, who are the key policymakers, how much power do they have to shape policy, and what is their current position towards PTB?	Stakeholder analysis
	In the research site being studied, how much priority does PTB currently receive on the health agenda?	Political prioritization analysis e.g., using the Shiffman and Smith framework for assessing the position of a health issue on the national policy agenda [25]
	How do material conditions in the research sites under study (e.g., physical safety, access to clean water, food supply) impact PTB outcomes?	Community-engaged participatory research, ethnography
	What are the most important PTB outcomes for people living within each research site, and what are their views on the optimal path forward for changing policies to affect those outcomes?	Community-engaged participatory research, ethnography
	What is the role of community advisory boards (CABs) in the policy making process? CABs are comprised of people with diverse characteristics who are linked by social ties, share common perspectives, and engage in joint action in geographical locations or settings. Involving them optimizes the potential for KTE [26, 27]	Qualitative methods
What strategies are associated with optimal KTE?	In the research sites under study, do evidence briefs for policymakers on preventing and treating PTB increase the likelihood that policies will be informed by the evidence?	Review of existing policy resource materials to examine how evidence briefs are used and whether they result in successful outcomes; case studies of examples of previous policymaker decision making, what evidence was used, and with what level of success (in the area of PTBI or parallel areas, e.g., HIV/AIDS); interventional studies that test whether evidence briefs affect policy decisions
	In the research sites under study, could an online “one stop shop” on evidence-based interventions for PTB increase the likelihood that policies will be informed by the evidence?	Landscape analyses of which resources currently exist, the availability of any repositories of information, policymaker preference and current use of tools to assure that this resource is useful and tailored to needs; interventional studies that test whether “one-stop shops” affect policy decisions
	In the research sites under study, could “deliberative dialogues” (Table 3) with policymakers on evidence-based interventions for PTB increase the likelihood that policies will be informed by the evidence?	Conduct a randomized study in which some sites are randomized to participate in a “deliberate dialogue” (control sites receive an evidence brief (Table 3) but do not participate in a dialogue about this brief)
	In the research sites under study, could “rapid response services” (Table 3) with policymakers on evidence-based interventions for PTB increase the likelihood that policies will be informed by the evidence?	Incorporate a rapid response service as part of the randomized study mentioned above
	In the research sites under study, could capacity building with policymakers on how to use evidence increase the likelihood that policies will be informed by the evidence?	Incorporate capacity building of policy makers as part of the randomized study
	In the research sites under study, could community engagement tools help policymakers to consider new perspectives?	Incorporate community engagement as part of the randomized study
	In the research sites under study, could the cultivation of learning collaboratives among policymakers on evidence-based interventions for PTB increase the likelihood that policies will be informed by the evidence?	Incorporate learning collaboratives as part of the randomized study
	What are the best ways to optimize the communication from CABs to policy makers? [26, 27] What is the role of advocacy groups?	Participant observation; key informant interviews with participants
What components of post-transfer engagement are associated with KTE strength and durability?	What is the duration of post-transfer engagement that is needed to support “stickiness” and sustainability of knowledge transfer?	Monitor and study research sites as part of the randomized study
	What levels of ongoing KTE support were required to achieve tangible policy change outcomes?	Process evaluation of the KTE efforts
	How might one improve KTE to create better sustainability in post-transfer engagement?	Exit interviews with participants in KTE efforts to assess “what worked” and “what didn’t”
Evaluation	Did policymakers use the evidence transferred? If they did use it, how did they use the evidence?	Qualitative key informant interviews of how evidence was used, and surveys of policymakers’ knowledge of scientific evidence pre/post KTE
	Did KTE efforts result in tangible changes in policies that promote improved PTB outcomes?	Case studies that track KTE from knowledge transfer to policy drafting and implementation to assess changes in funding levels, regulations, etc.
	Do KTE efforts, when they have successfully informed policymaking, have a measureable impact on PTB health outcomes?	Natural experiments, ideally using comparison sites, to track PTB outcomes before and after evidence-informed policies were implemented

We conclude that an important early priority is to gather baseline information across research sites being studied on: 1) the current “evidence” sources that policymakers already use, 2) current policy efforts at tackling PTB that are already in place, 3) existing perceptions and misperceptions of the root causes of PTB held by policymakers, consumers, and providers, 4) prior successes with decreasing PTB, and 5) the readiness of policymakers to review evidence of effective practices. Qualitative methods, including in-depth interviews and focus groups, are best suited to capture these baseline data, as well as content analyses of existing policies and protocols. Case study designs will further provide opportunities to understand the complex context of political, cultural and economic conditions in a holistic way, while integrating what is already known through previous research and using between-site comparisons. This “landscaping” should also track preliminary specific areas of concern and interest to policymakers prior to attempting any interventions.

Once the context and strategies of KTE are understood, researchers should then focus on evaluation of the effectiveness of a KTE strategy. To understand how a KTE strategy has been previously applied, retrospective designs may work well. The advantage of this type of study is that it provides an opportunity to explore in-depth the implementation of a strategy of interest. Randomized controlled trials, the “gold standard” in research, may be difficult to conduct at a policy level. Randomization can be helpful if there are enough settings in which the policy strategy is being considered to warrant this design. When a policy strategy is being rolled out on a broad level—across a country for example—KTE strategies could be applied differentially across local policy decision-making units (e.g., at the county or municipal level) and the processes and outcomes studied and compared. The advantage of well-designed randomized studies is that they have more power to causally associate a KTE strategy with a policy change, as opposed to changes that resulted from other factors present in the social, political and economic context.

Conducting an RCT of a policy intervention at the national level is typically very costly. RCTs would also require intensive policymaker and citizen engagement (e.g., discussion of evidence, engagement regarding which policy options to implement, and provision of technical assistance as policies are operationalized and implemented and roll out of the policy in an ethical manner). RCTs are probably most practical when sufficient resources are available. For example, with adequate funding, an RCT would be a valuable design to test a “one-stop shop” of information on PTB, a rapid response team to provide rapid provision of evidence on PTB when policymakers request it, and twinning policymakers with local technical assistance providers. Such a trial could help to ascertain the “dosage” of policy maker engagement to test the question of KTE sustainability.

## Conclusion

In this article, we have identified and characterized a set of important research questions related to KTE with policymakers that could potentially be used to reduce the global burden of PTB. We have also proposed some study ideas and strategies that hold promise for the field. Discovering the right strategies, or bundles of strategies, to optimize KTE for this group of stakeholders is crucial for the success of the PTBI. We hope that our proposed research agenda stimulates further debate and discussion on research priorities to soon bend the curve of PTB mortality.

## Additional file

Additional file 1: Methods for synthesizing the literature on KTE and summary of the evidence. (DOCX 17 kb)
