# Supplementary material for: Reducing the global burden of Preterm Birth through knowledge transfer and exchange: a research agenda for engaging effectively with policymakers
Source: Reprod Health. 2016 Mar 18;13:26. doi: 10.1186/s12978-016-0146-8 (PMC4797256; doi:10.1186/s12978-016-0146-8)
Supplement: Additional file 1: — Methods for synthesizing the literature on KTE and summary of the evidence. (DOCX 17 kb) [file 12978_2016_146_MOESM1_ESM.docx]

**Supporting Information File S1**

**Methods for synthesizing the research literature on knowledge transfer and exchange (KTE)**

We conducted searches of the scientific literature on August 31, 2014 in an effort to identify systematic and narrative reviews of efficacious KTE strategies, and ideally “systematic reviews of systematic reviews” describing such strategies.

Using a range of relevant terms, we searched titles, abstracts and keywords in PubMed and the Cochrane Database of Systematic Reviews. The latter resource includes the Database of Abstracts of Reviews of Effectiveness (DARE). The Health Systems Evidence database at McMaster University was also searched.

Search terms included the following, in appropriate combinations:

- Systematic review OR review OR Cochrane Database of Systematic Reviews
- (Knowledge OR information) AND (transfer* OR translat* OR uptake OR action OR integrat* OR implement* OR disseminat*)
- Preterm birth OR prematurity OR “maternal health” OR “maternal and child health” OR (mother* and child*) OR “women’s health” OR “child health” OR pregnancy OR neonat* OR (clinical AND practice) OR (health* AND policy) OR (lay* OR consumer*)

We screened search results (n= 775) and identified reviews for inclusion (n=16 [1-16]).

The identified reviews fell generally into two categories:

1. Reviews of KTE strategies, irrespective of the health care intervention context in which they had been applied (“Cross-cutting KTE”), n=7 [1-7]; and
2. Reviews of KTE strategies applied specifically to maternal, neonatal and child health (MNCH) interventions (“MNCH KTE”), n=9 [10-16].

Cross-cutting KTE reviews considered the efficacy of specific KTE strategies in any area of health care—for example, “decision aids” for people facing health treatment or screening decisions. MNCH KTE reviews considered the effectiveness of specific KTE strategies in health care contexts specifically relevant to the PTBI—for example, “decision aids” to improve care for pregnant women.

We extracted from each included review its citation information and scope, key characteristics of included primary studies (or included reviews) and its key findings. We synthesized evidence from these findings primarily in tabular form, organized by stakeholder type.

**Key cross-cutting KTE review:** One “review of reviews,” by Grimshaw and colleagues, provided an enhanced understanding of “what works” in KTE strategies across all health care interventions [2]. In addition to describing key concepts in KTE, this work provided a summary overview of cross-cutting KTE strategies. Grimshaw and colleagues examined Cochrane reviews of KTE strategies for health care providers as well as health care “consumers,” i.e., individuals and their families. Most of these reviews included multiple randomized controlled trials. In examining the evidence for KTE strategies addressing individuals and providers, Grimshaw and colleagues only considered Cochrane reviews. However, given the variable methodological quality of non-Cochrane reviews and the consistently high quality of Cochrane reviews, this was judged by the Working Group to be an appropriate resource. Grimshaw and colleagues also analyzed the Supporting Policy-Relevant Reviews and Trials (SUPPORT) tools for facilitating KTE with policy-makers [17].

**Key MNCH KTE reviews:** Several systematic reviews (including reviews of reviews) directly addressed KTE strategies for improving MNCH outcomes, although none directly addressed KTE strategies for improving outcomes relevant to preterm birth. The most useful review of reviews was one by Nair and colleagues focused on barriers to and facilitators of care in the context of MNCH interventions [12].

**References:**

1. Giugliani C, Harzheim E, Duncan MS, et al. Effectiveness of community health workers in Brazil: a systematic review. J Ambul Care Manage 2011;34:326–38.
2. Grimshaw JM, Eccles MP, Lavis JN, Hill SJ, Squires JE. Knowledge translation of research findings. Implement Sci. 2012 May 31;7:50.
3. McCormack L, Sheridan S, Lewis M, Boudewyns V, Melvin CL, Kistler C, et al. Communication and dissemination strategies to facilitate the use of health-related evidence. Evid Rep Technol Assess (Full Rep). 2013 Nov;(213):1-520.
4. Oliver K, Innvar S, Lorenc T, Woodman J, Thomas J. A systematic review of barriers to and facilitators of the use of evidence by policy-makers. BMC Health Serv Res. 2014 Jan 3;14:2.
5. Urquhart C, Currell R, Grant Maria J, et al. Nursing record systems: effects on nursing practice and health care outcomes. Cochrane Database Syst Rev 2009;(1):CD002099.
6. Murthy L, Shepperd S, Clarke MJ, Garner SE, Lavis JN, Perrier L, Roberts NW, Straus SE. Interventions to improve the use of systematic reviews in decision-making by health system managers, policy-makers and clinicians. Cochrane Database Syst Rev. 2012 Sep 12;9: CD009401.
7. Haws RA, Thomas AL, Bhutta ZA, et al. Impact of packaged interventions on neonatal health: a review of the evidence. Health Policy Plan 2007;22:193–215.
8. Ibanez G, de Reynal de Saint Michel C, Denantes M, et al. Systematic review and meta-analysis of randomized controlled trials evaluating primary care-based interventions to promote breastfeeding in low-income women. Fam Pract 2012;29:245–54.
9. Kongnyuy EJ, Uthman OA. Use of criterion-based clinical audit to improve the quality of obstetric care: a systematic review. Acta Obstet Gynecol Scand 2009;88:873–81
10. Lassi ZS, Haider BA, Bhutta ZA. Community-based intervention packages for reducing maternal and neonatal morbidity and mortality and improving neonatal outcomes. Cochrane Database Syst Rev 2010;(11):CD007754.
11. Marston C, Renedo A, McGowan CR, et al. Effects of community participation on improving uptake of skilled care for maternal and newborn health: a systematic review. PLoS ONE 2013;8:e55012.
12. Nair M, Yoshida S, Lambrechts T, Boschi-Pinto C, Bose K, Mason EM, Mathai M. Facilitators and barriers to quality of care in maternal, newborn and child health: a global situational analysis through metareview. BMJ Open. 2014 May 22;4(5):e004749.
13. Opiyo N, English M. In-service training for health professionals to improve care of the seriously ill newborn or child in low and middle-income countries (Review). Cochrane Database Syst Rev. 2010 Apr 14;(4):CD007071.
14. Oyo-Ita A, Nwachukwu Chukwuemeka E, et al. Interventions for improving coverage of child immunization in low- and middle-income countries. Cochrane Database Syst Rev 2011;(7):CD008145.
15. Glenton C, Scheel IB, Lewin S, et al. Can lay health workers increase the uptake of childhood immunisation? Systematic review and typology. Trop Med Int Health 2011;16:1044–53.
16. Vlemmix F, Warendorf JK, Rosman AN, Kok M, Mol BW, Morris JM, Nassar N. Decision aids to improve informed decision-making in pregnancy care: a systematic review. BJOG. 2013 Feb;120(3):257-66.
17. Lavis JN, Oxman AD, Lewin S, Fretheim A. SUPPORT Tools for evidence-informed health Policy making (STP). Health Res Policy Syst. 2009 Dec 16;7 Suppl 1:I1.
